# Long-term changes in overjet in individuals with and without orthodontic treatment: A 30-year cohort study

**DOI:** 10.1007/s00056-025-00633-7

**Published:** 2026-01-08

**Authors:** Jolina Bokan, Jonas Q. Schmid, Claudius Middelberg, Moritz Kanemeier, Sebastian-Edgar Baumeister, Ulrike Ehmer, Ariane Hohoff, Thomas Stamm

**Affiliations:** 1 Department of Orthodontics, University of Münster, Münster, Germany; 2 Institute of Health Services Research in Dentistry, University of Münster, Münster, Germany

**Keywords:** Malocclusion, Oral health-related quality of life, DMFT index, Longitudinal cohort, Dental trauma, Malokklusion, Mundgesundheitsbezogene Lebensqualität, DMFT-Index, Longitudinale Kohorte, Zahnverletzungen

## Abstract

**Purpose:**

Large overjet increases the risk of traumatic dental injury (TDI). This study, part of the Münster Long-Term Study (MLS), aimed to investigate long-term changes in overjet with or without orthodontic treatment and to evaluate whether correcting large overjet can reduce Decayed, Missing, and Filled Teeth Index (DMFT) scores of maxillary incisors.

**Methods:**

Eligible for inclusion in this longitudinal cohort study (MLS) were 667 primary school children who underwent two systematic orthodontic examinations between 1981 and 2001 (T0). Data collection at follow-up in 2022–2023 (T1) included a clinical examination and intraoral scans to evaluate overjet, DMFT, TDI and orthodontic treatment history. Participants were divided into treated and untreated groups. Statistical analysis included Mann–Whitney U tests and paired t‑tests, with significance set at α = 0.05.

**Results:**

A total of 73 participants could be enrolled in the follow-up: 50 treated (female/male: 27/23; mean age 38.3 ± 5.1 years) and 23 untreated (female/male: 10/13; mean age 39.1 ± 6.0 years). The mean observation period was 30.0 ± 5.3 years. At T0, the mean overjet was significantly larger in the treated group than in the untreated group (4.0 ± 1.78 mm vs 2.6 mm ± 0.79, *p* = 0.002). The treated group showed a significant reduction in overjet from T0 to T1 (*p* < 0.001), while changes in the untreated group were not significant (*p* = 0.601). At T1, differences in overjet between groups were no longer significant (*p* = 0.105) and there was no significant difference in DMFT scores of the maxillary incisors (*p* = 0.276). TDI of the anterior teeth occurred in 30.0% (*n* = 15) of the treated group and in 8.7% (*n* = 2) of the untreated group at T1. Logistic regression analysis showed that each millimeter increase in overjet at T0 was associated with 47% higher odds of TDI (odds ratio 1.47, 95% CI 1.05–2.13).

**Conclusion:**

Orthodontic treatment can effectively improve overjet in the long-term. However, it did not reduce DMFT scores of the maxillary incisors in this MLS sample.

## Abstract (de)

**Zielsetzung:**

Ein vergrößerter Overjet erhöht das Risiko für traumatische Zahnverletzungen (TDI). Diese Studie, die Teil der Münsteraner-Langzeitstudie (MLS) ist, hatte zum Ziel, langfristige Veränderungen des Overjets mit oder ohne kieferorthopädische Behandlung zu untersuchen sowie zu evaluieren, ob die Korrektur eines vergrößerten Overjets den DMFT („decayed, missing, and filled teeth index“) der oberen Schneidezähne senken kann.

**Methoden:**

Für die Aufnahme in diese longitudinale Kohortenstudie (MLS) waren 667 Grundschulkinder geeignet, die zwischen 1981 und 2001 (T0) zwei systematische kieferorthopädische Untersuchungen erhalten hatten. Die Datenerhebung bei der Nachuntersuchung in den Jahren 2022–2023 (T1) umfasste eine klinische Untersuchung und intraorale Scans zur Beurteilung von Overjet, DMFT, TDI sowie eine Evaluation der kieferorthopädischen Behandlungshistorie. Die Teilnehmenden wurden in Gruppen mit kieferorthopädischer Behandlung und ohne Behandlung aufgeteilt. Die statistische Analyse umfasste Mann-Whitney-U-Tests und gepaarte t‑Tests, wobei die Signifikanz auf α = 0,05 festgelegt wurde.

**Ergebnisse:**

Insgesamt konnten 73 Teilnehmende in die Nachuntersuchung eingeschlossen werden: 50 behandelte (weiblich/männlich: 27/23; Durchschnittsalter 38,3 ± 5,1 Jahre) und 23 unbehandelte (weiblich/männlich: 10/13; Durchschnittsalter 39,1 ± 6,0 Jahre). Die mittlere Beobachtungsdauer betrug 30,0 ± 5,3 Jahre. Zum Zeitpunkt T0 war der mittlere Overjet in der behandelten Gruppe signifikant größer als in der unbehandelten Gruppe (4,0 ± 1,78 vs. 2,6 ± 0,79 mm, *p* = 0,002). Die behandelte Gruppe zeigte eine signifikante Reduktion des Overjets von T0 zu T1 (*p* < 0,001), während sich in der unbehandelten Gruppe keine signifikanten Veränderungen zeigten (*p* = 0,601). Zum Zeitpunkt T1 waren die Unterschiede im Overjet zwischen den Gruppen nicht mehr signifikant (*p* = 0,105), und es gab keinen signifikanten Unterschied in den DMFT-Werten der oberen Schneidezähne (*p* = 0,276). Eine TDI der Frontzähne trat bei 30,0 % (*n* = 15) der behandelten Gruppe und bei 8,7 % (*n* = 2) der unbehandelten Gruppe zum Zeitpunkt T1 auf. Die logistische Regressionsanalyse zeigte, dass jeder Millimeter Anstieg des Overjet bei T0 mit um 47 % größeren Odds für eine TDI verbunden war (Odds Ratio 1,47, 95 %-KI 1,05–2,13).

**Schlussfolgerung:**

Eine kieferorthopädische Behandlung kann den Overjet langfristig signifikant verbessern. In dieser MLS-Kohorte zeigte sich jedoch kein Effekt auf den DMFT-Index der oberen Schneidezähne.

## Introduction

Large overjet is a common malocclusion with dental or skeletal causes, often involving mandibular deficiency, maxillary excess, or both. The upper incisors are characteristically prominent, and the lower lip may catch behind them [4, 32]. Globally, it affects approximately 23% of individuals in mixed dentition and 20% in permanent dentition [1].

Large overjet increases the risk of bullying [38, 39] and negatively affects oral health-related quality of life (OHRQoL) [23, 42]. Furthermore, it significantly raises the risk of traumatic dental injuries (TDIs) [3, 30, 31, 36], potentially accounting for over 200 million TDIs worldwide [36]. Given the high prevalence of TDIs and the financial burden of treatment, prevention is essential [3, 36]. Orthodontic overjet correction appears to effectively reduce this risk [4]. Large overjet also correlates with other malocclusion traits [20] and potentially increases the long-term risk of tooth loss [33].

Currently, there is a lack of evidence on the long-term oral health benefits of overjet correction and orthodontic treatment [31]. Public health institutions have emphasized the need for research in this area [22, 30, 44]. Although several studies have focused on overjet changes during early dentition phases, longitudinal research extending into adulthood is limited [35]. Previous studies showed variable outcomes, indicating a lack of consensus in the literature. Some studies report a slight long-term decrease in overjet among untreated adolescents with normal occlusion [16, 45], while one study observed an increase [35]. In untreated adults, overjet generally remains stable [6, 9, 15]. In untreated adolescents with severe overjet, most studies found no improvement into mid-adulthood [18, 35], although one study reported some improvement [24]. However, no previous study has included oral health outcomes.

To address these questions, the Münster Long-Term Study (MLS) was established—a cohort of individuals originally assessed in childhood and followed up three decades later.

Therefore, the aim of the present study was to investigate long-term changes in overjet with or without orthodontic treatment and to evaluate whether correcting large overjet can reduce TDIs and Decayed, Missing, and Filled Teeth Index (DMFT) scores of maxillary incisors. The null hypothesis was tested that there are no statistically significant differences in long-term overjet changes between individuals with and without orthodontic treatment.

## Patients and methods

This analysis was undertaken as part of the MLS and received approval from the local ethics committee of the Medical Faculty of the University of Münster, Germany (2020-407-f-S). The study was reported according to the Strengthening the Reporting of Observational Studies in Epidemiology (STROBE) guidelines [12]. Data collection and analysis were conducted at the Department of Orthodontics, University Hospital Münster, Germany. The follow-up-examinations were performed by one calibrated examiner from February 2022 to November 2023.

### Study design and participants

Eligible for inclusion in this study were children from local primary school classes who participated in a systematic orthodontic examination at the Department of Orthodontics, University Hospital Münster, Germany between 1981 and 2001 (T0). The participants received annual clinical examinations, impressions for plaster models, a bite registration in habitual intercuspation and a set of standardized extraoral and intraoral photographs. Parents were instructed to consult an orthodontist of their choice if orthodontic treatment was indicated at that time.

The criteria for inclusion in the presented longitudinal follow-up (T1) were (1) participation in at least two systematic early phase orthodontic examinations between 1981 and 2001, (2) presence of plaster casts, (3) informed consent to participate. Exclusion criteria were (1) participation in less than two systemic early phase orthodontic examinations, (2) missing plaster casts, (3) missing first molars or permanent central incisors, (4) no consent to participate, (5) orofacial syndromes and clefts, and (6) presence of current orthodontic treatment. If multiple models at T0 were available, the earliest one with presence of first molars and permanent central incisors was chosen.

After follow-up (T1), the cohort was divided into two groups: an untreated group with no history of orthodontic treatment, and a treated group that had undergone orthodontic treatment between T0 and T1.

### Data collection

Participants were contacted by post and invited to participate in the follow-up.

Data collection at T1 included the following: (1) a questionnaire to assess history of orthodontic treatment and TDIs, (2) a clinical examination to register DMFT scores as proposed by the World Health Organization (WHO) [47], and (3) intraoral scans using a Trios 3 scanner (3Shape, Copenhagen, Denmark).

Plaster models from T0 were digitized and stereolithography (STL) files of T0 and T1 models were imported into the Blender Software 4.0 (Blender Foundation, Amsterdam, Netherlands) which was used for measurements. Overjet on digital models was determined similar to the SHIP studies and later categorized as mild (> 0 to < 4 mm), moderate (4–6 mm), or severe (> 6 mm) [5, 21].

### Statistical analysis

Descriptive statistics were calculated for all variables and reported as mean, standard deviation (SD), minimum and maximum value. To assess the normality of the data distribution, Shapiro–Wilk tests were used. Nonparametric tests were used if the data were not normally distributed (*p* < 0.05).

Chi-square tests and Mann–Whitney U tests were used to evaluate differences in the baseline characteristics. Mann–Whitney U tests were used to assess intergroup differences of overjet and DMFT scores, paired t‑tests were used to assess intragroup differences for overjet changes. Logistic regression analysis was performed to assess whether the risk of TDI was associated with overjet at T0. The significance level was set to α = 5%, and a *p*-value < 0.05 was considered significant.

Intrarater reliability for the measurements was evaluated using intraclass correlation coefficients (ICC). For this purpose, 10% of the cases were randomly selected using a random number generator and were re-evaluated by the same calibrated examiner after at least 4 weeks. ICC estimates were calculated based on a single measurement, absolute-agreement, 2‑way mixed effects model. ICC values were interpreted according to Koo and Li [28].

All statistical analyses were conducted using R 4.4.2 (R Foundation, Vienna, Austria).

## Results

### Intrarater reliability

Intrarater reliability was excellent for overjet and good for DMFT and maxillary incisors DMFT (Table 1).

**Table 1:** Measurements and intrarater reliability Messungen und Intrarater-Reliabilität

Variable	Description	Reliability
Overjet	The horizontal overlap of the upper to lower permanent front teeth in millimeters measured at the most severe site	0.997^a^
Overall DMFT	Decayed, Missing, and Filled Teeth Index as described by WHO [47] is based on a total of 32 permanent teeth (18–48)	0.897^a^
Maxillary incisors DMFT	Decayed, Missing, and Filled Teeth Index analyzed exclusively for the maxillary incisors (12–22)	0.881^a^

^a^ Koo and Li [28]: ICC < 0.5: poor reliability; 0.5 ≤ ICC < 0.75: moderate reliability; 0.75 ≤ ICC < 0.9: good reliability; ICC ≥ 0.9: excellent reliability

### Sample characteristics

**Fig. 1:**
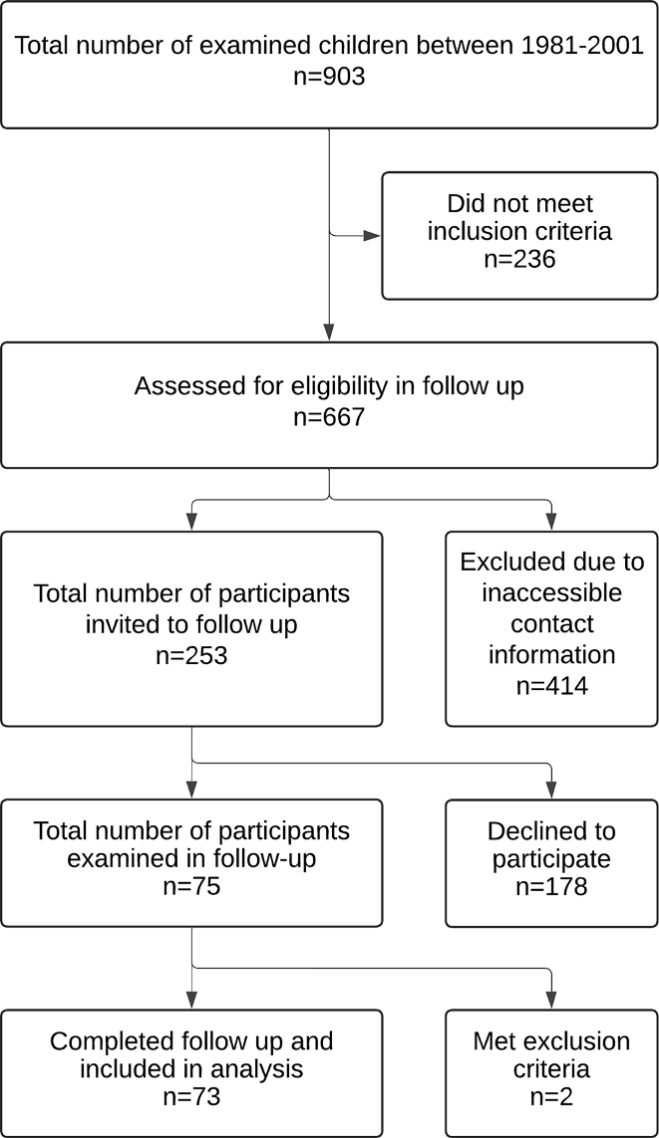
STROBE flow diagram of participants STROBE Flussdiagramm der Teilnehmer

The participant flow is illustrated in Fig. 1. The initial MLS sample consisted of 903 children who participated in at least one systematic orthodontic examination at T0. Overall, 667 subjects met the inclusion criteria for re-examination at T1. However, due to the long observation period, current contact information could only be obtained for 253 subjects. The response rate for participation in this study was 29.6%, with 75 subjects finally accepting our invitation and all giving written informed consent to participate in the re-examination at T1. A total of 2 individuals met the exclusion criteria, leading to a final sample of 73 participants (female/male: 37/36; mean age 38.6 ± 5.3 years).

Baseline characteristics of participants at T0 and T1 are presented in Table 2. The mean observation period from T0 to T1 was 30.0 ± 5.3 years. The untreated group consisted of *n* = 23 participants (female/male: 10/13; mean age 39.1 ± 6.0 years), the treated group consisted of *n* = 50 participants (female/male: 27/23; mean age 38.3 ± 5.1 years). Gender was almost equally distributed in the study population (Table 2). However, the comparison of variables between genders showed a statistically significant difference in age at baseline (T0), with females being slightly younger than males (*p* = 0.046). No significant gender differences were observed in other variables.

**Table 2:** Characteristics of participants at baseline (T0) and follow-up (T1) Merkmale der Probanden zu Beginn (T0) und beim Follow-up (T1)

Characteristics	Full sample (*n* = 73)	Treated (*n* = 50)	Untreated (*n* = 23)	*p* value
*Gender, n (%)*				0.404^b^
Female	37 (50.7)	27 (54.0)	10 (43.5)	
Male	36 (49.3)	23 (46.0)	13 (56.5)	
*Age T0 (years)*				0.764^a^
Mean ± SD	8.1 ± 1.0	8.1 ± 1.0	8.1 ± 1.0	
Min–Max	6–11	6–10	7–11	
*Age T1 (years)*				0.720^a^
Mean ± SD	38.6 ± 5.3	38.3 ± 5.1	39.1 ± 6.0	
Min–Max	31–48	31–48	31–48	
*Observation period T1–T0 (years)*				0.708^a^
Mean ± SD	30.0 ± 5.3	29.8 ± 5.1	30.5 ± 5.9	
Min–Max	22–39	22–39	22–39	
*Orthodontic treatment n (%)*				
No	23 (31.5)	0 (0.0)	23 (100.0)	–
Yes	50 (68.5) 13 (17.8) 3 (4.1) 34 (46.6)	50 (100.0)	0 (0.0)	
Removable appliance		13 (26.0)	–	
Fixed appliance		3 (6.0)	–	
Both removable and fixed appliance		34 (68.0)	–	

*SD* standard deviation, *Min* minimum, *Max* maximum

^a^ Mann–Whitney U test

^b^ Pearson Chi-square test

### Overjet changes

**Fig. 2:**
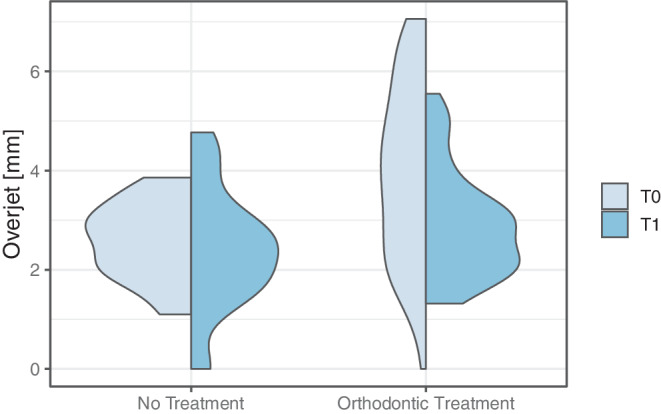
Split violin plot representing the overjet reduction from T0 to T1 Violinendiagramm zur Darstellung der Verringerung des Overjets von T0 zu T1

**Fig. 3:**
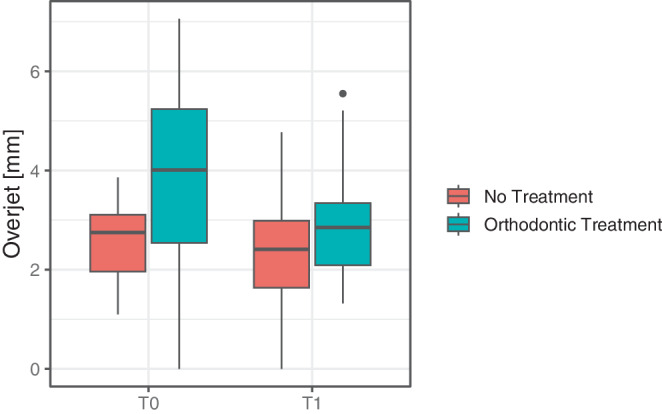
Boxplots representing intergroup differences of overjet at T0 and T1 Boxplots zur Darstellung der Unterschiede zwischen den Gruppen hinsichtlich des Overjets zu den Zeitpunkten T0 und T1

Overjet measurements are presented in Table 3 and Figs. 2 and 3. Prior to treatment (T0), 48.0% of the participants in the treated group had a mild overjet, 30.0% had a moderate overjet, and 22.0% had a severe overjet. In contrast, all individuals in the untreated group presented a mild overjet at T0 (Fig. 3). The mean overjet in the untreated group was 2.58 ± 0.79 mm compared to 3.95 ± 1.78 mm in the treated group; this difference was statistically significant (*p* = 0.002, Table 3).

At T1, mild overjet was almost as common in the treated as in the untreated group (Fig. 2). The overjet difference between the untreated and treated participants was no longer significant (2.39 ± 1.23 mm vs. 2.89 ± 1.06 mm, *p* = 0.105, Table 3). While the treated group experienced a significant overjet reduction from T0 to T1 (0.92 ± 1.77 mm, *p* < 0.001, Table 3), the overjet changes in the untreated group were not significant (*p* = 0.601).

**Table 3:** Intra- and intergroup comparison of overjet [mm] Intra- und Intergruppenvergleich des Overjet [mm]

	T0			T1			∆T0–T1			Intragroup test	
	Mean ± SD	Min–Max	95% CI	Mean ± SD	Min–Max	95% CI	Mean ± SD	Min–Max	95% CI		95% CI
Untreated	2.58 ± 0.79	1.10–3.86	[2.22; 2.94]	2.39 ± 1.23	0.00–4.77	[1.84; 2.94]	0.63 ± 1.81	−2.60 to 4.43	[−0.15; 1.41]	*p* = 0.601	[−0.49; 0.83]
Treated	3.95 ± 1.78	0.00–7.06	[3.44; 4.46]	2.89 ± 1.06	1.32–5.55	[2.58; 3.20]	0.92 ± 1.77	−3.84 to 5.06	[0.41; 1.43]	*p* < 0.001	[0.54; 1.51]
Intergroup test	*p* = 0.002			*p* = 0.105			*p* = 0.357			*–*	*–*

*SD* standard deviation, *Min* minimum, *Max* maximum, *95% CI* 95% confidence interval

### DMFT findings

**Fig. 4:**
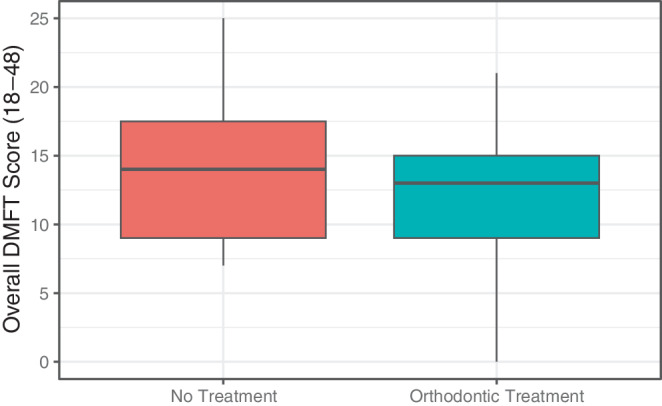
Boxplots representing intergroup differences of overall DMFT Index scores at T1 Boxplots zur Darstellung der Unterschiede zwischen den Gruppen hinsichtlich der DMFT-Gesamtwerte zum Zeitpunkt T1

At T1, the treated group had slightly lower mean overall DMFT scores (teeth 18–48) of 12.14 ± 5.14 compared to the untreated group of 13.87 ± 5.50 (Table 4; Fig. 4). This difference was not statistically significant (*p* = 0.380), but it is noteworthy that the minimum values for DMFT scores were higher in the untreated group. Looking at the DMFT components, both groups had the same mean of decayed teeth (untreated 0.74 ± 1.57; treated: 0.74 ± 1.41). The treated group exhibited a marginally higher mean number of missing teeth (3.46 ± 1.97) compared to the untreated group (3.09 ± 1.56). Conversely, the treated group maintained fewer restored teeth with a mean of 8.30 ± 5.36 compared to 10.04 ± 5.10 in the untreated group.

### Trauma findings

Although TDI of the anterior teeth occurred in 30.0% (*n* = 15) of the treated group and only in 8.7% (*n* = 2) of the untreated group at T1, the DMFT scores of the maxillary incisors were not significantly different between groups (*p* = 0.276, Table 4).

**Fig. 5:**
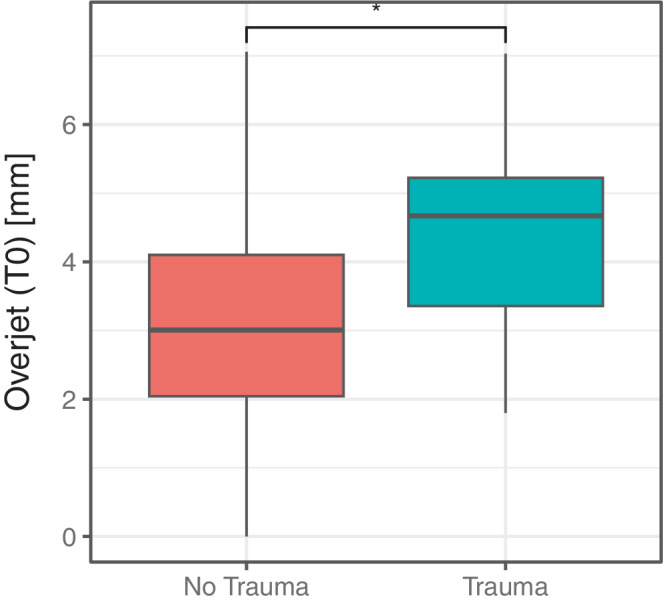
Boxplot for association between traumatic dental injury (TDI) history and overjet at T0 Boxplot zum Zusammenhang zwischen traumatischen Zahnverletzungen (TDI) in der Anamnese und Overjet zum Zeitpunkt T0

Logistic regression analysis showed that the risk of TDI was associated with overjet at T0. The odds ratio (OR) for trauma was 1.47 (95% CI 1.05–2.13) indicating that for each millimeter increase in overjet, the odds of experiencing trauma increased by 47%. This finding was statistically significant (*p* = 0.029, Fig. 5).

**Table 4:** Intergroup comparison of DMFT scores at T1 Intergruppenvergleich der DMFT-Werte zum Zeitpunkt T1

	Overall DMFT			Maxillary Incisors DMFT		
	Mean ± SD	Min–Max	95% CI	Mean ± SD	Min–Max	95% CI
Untreated	13.87 ± 5.50	7.00–25.00	[11.49; 16.25]	0.11 ± 0.32	0.00–1.00	[−0.05; 0.26]
Treated	12.14 ± 5.14	0.00–21.00	[10.67; 13.62]	0.27 ± 0.54	0.00–2.00	[0.12; 0.43]
Intergroup test	*p* = 0.380			*p* = 0.276		

*SD* standard deviation, *Min* minimum, *Max* maximum, *95% CI* 95% confidence interval, *DMFT* Decayed, Missing, and Filled Teeth Index

## Discussion

The present study used longitudinal data from the MLS to evaluate long-term changes in overjet over a 30-year period with and without orthodontic treatment, and to evaluate whether correcting large overjet can reduce DMFT scores of maxillary incisors. The results indicate that orthodontic treatment did significantly reduce overjet in the long term, but did not reduce DMFT scores of maxillary incisors in this cohort. Overjet changes in untreated patients were not significant. The odds for dental trauma increased with larger overjet at baseline.

The reduction in overjet among the treated group can be attributed to orthodontic interventions [4]. The difference in age at T0 by gender is likely to be coincidental, as the cohort was drawn from school classes, ensuring similar ages across the board. The lack of significant improvement in DMFT scores suggests that while orthodontic treatment effectively addresses malocclusion and overjet, it may not directly influence caries risk, possibly due to factors like nutrition, oral hygiene practices, and fluoride exposure, which were not controlled for in this study. The increased risk of traumatic dental injuries with larger overjet could be due to the prominence of maxillary incisors and inadequate lip coverage, making them more susceptible to TDI [31].

At baseline, 24.7% of the 73 participants had an overjet of 4–6 mm and 13.7% had an overjet of > 6 mm, indicating a high prevalence of large overjet in this north-western region of Germany. These values are higher than those reported in the NHANES III study, which found 5–6 mm in 18.9% and ≥ 7 mm in 3.6% of 8‑ to 11-year-olds in the United States [37], but slightly lower than the 20.3% with an overjet of ≥ 6 mm found among north-eastern German schoolchildren [19]. The pattern also mirrors a recent 20-year regional survey from western Germany, where increased overjet (> 6 mm) was the most frequent finding (24.3%) [27]. These results confirm that there are regional differences in the prevalence of class II malocclusion and large overjet, with a higher prevalence observed in Europe [1].

There is a lack of consensus in the literature on the long-term changes of large overjet in untreated patients. Previous studies showed variable outcomes [13, 14, 18, 24, 35]. The present study identified a mean overjet reduction of 0.63 mm in the untreated subjects with initial overjet < 4 mm, which is in good agreement with previous literature [16, 45]. However, these overjet changes were not statistically significant, and the clinical implications of these findings should be interpreted with caution.

The detected overjet reduction among the treated group was higher with a mean of 0.92 mm. This reduction was statistically significant. Although the extent of the overjet reduction seems limited, it can be stated that orthodontic treatment did significantly reduce overjet in the long term. This is consistent with previous findings, indicating an effective overjet reduction with treatment and no significant group differences between treated and untreated at follow-up [14, 24].

The finding that 80.8% of adults at follow-up showed an overjet of > 0 < 4 mm aligns closely with US findings, where 80.7% had an overjet of 1–4 mm [37], and with Dutch data, which reported 74–78% for an overjet of 0–4 mm [8].

An 18-year longitudinal study provided a comparative analysis of treated severe class II malocclusions [7]. Their findings indicated that individuals who received treatment for class II malocclusion exhibited reduced oral health impairment compared to the general German population, as reflected in DMS 5 data, where the mean DMFT was reported to be 11.2 for the 35–44 age cohort, both excluding third molars [7, 26]. Within the context of the current investigation, mean DMFT values excluding third molars were higher (untreated: 10.7; treated: 9.0) than those reported by Bock et al. [7]. However, the data indicate the same tendency: the DMFT scores of the orthodontically treated group were significantly lower than those of the DMS 5 population-representative sample.

The latest German population-representative study (DMS 6) was conducted between October 2022 and July 2023 [34] and reported a mean DMFT of 8.3 in the same age group [25] also excluding third molars [29]. The DMS 6 clinical dental examinations were conducted in fieldwork settings under suboptimal lighting and in absence of air drying, which could make the identification of tooth-colored restorations challenging. With limited interrater reliability in detecting carious lesions [34] and the exclusion of restorations related to molar incisor hypomineralization (MIH) or dental trauma [25], a direct comparison of DMFT values to the present results seems difficult.

Although the prevalence of TDIs in this sample (23.3%) was consistent with previous studies [4, 36], it is noteworthy that 15 TDIs (30.0%) occurred in the treated group and only two (8.7%) in the untreated group. However, there were no statistically significant differences in maxillary incisor DMFT scores. This finding is consistent with existing literature: Helm and Petersen (1989) also found higher DMFS values in the maxillary incisors of participants with increased overjet, but these were not significant either. Overall, they found no association between orthodontic treatment and a reduced caries experience in adults [17]. This was also demonstrated in other longitudinal studies [11, 46]. Population-based studies have recently clarified that, while orthodontic treatment appears to reduce the risk of carious lesions in later life, it also correlates with a higher incidence of filled teeth. Therefore, no overall risk-reducing effect on DFT/DMFT could be demonstrated [2, 10].

### Strengths and limitations

The main strength of this study is the long-term follow-up of 30 years, providing important longitudinal data from both orthodontically untreated and treated subjects over a substantial period. Despite the high nonresponse rate, it must be seen as a great achievement to re-engage patients after 30 years.

However, some methodological limitations must be considered when interpreting the results. First, the study is susceptible to nonresponse bias, as respondents may demonstrate higher dental awareness [43]. Second, information about orthodontic treatment history was collected many years later. The possibility of early orthodontic treatment prior to T0 could not be completely ruled out, which could explain the absence of negative overjet in this sample. Finally, socioeconomic status was not addressed to eliminate possible inequalities in parental income or education.

It is only in adulthood that the benefits concerning dental health are realized; consequently, the effectiveness of treatment should be evaluated over a prolonged period of time [41], which underscores the necessity for longitudinal comparative cohort studies [30, 40].

## Conclusion

Orthodontic treatment can effectively improve overjet in the long-term. However, it did not reduce Decayed, Missing, and Filled Teeth (DMFT) Index scores of the maxillary incisors in this MLS sample.

## Data Availability

The datasets used in the current study are available from the corresponding author on reasonable request.
